# Retrospective validation of whole genome sequencing-enhanced surveillance of listeriosis in Europe, 2010 to 2015

**DOI:** 10.2807/1560-7917.ES.2018.23.33.1700798

**Published:** 2018-08-16

**Authors:** Ivo Van Walle, Jonas Torgny Björkman, Martin Cormican, Timothy Dallman, Joël Mossong, Alexandra Moura, Ariane Pietzka, Werner Ruppitsch, Johanna Takkinen, European Listeria WGS typing group

**Affiliations:** 1 European Centre for Disease Prevention and Control (ECDC), Stockholm, Sweden; 2 Statens Serum Institut, Copenhagen, Denmark; 3 National University of Ireland Galway, Galway, Ireland; 4 Public Health England, London, United Kingdom; 5 Laboratoire national de santé, Dudelange, Luxembourg; 6 Institut Pasteur, National Reference Center and WHO Collaborating Center Listeria, Biology of Infection Unit, Inserm U1117, Paris, France; 7 Österreichische Agentur für Gesundheit und Ernährungssicherheit, Graz/Vienna, Austria; 8 The European Listeria WGS typing group members have been listed at the end of this article

**Keywords:** epidemiology, food-borne infections, laboratory surveillance, Listeria, public health policy, whole genome sequencing, WGS

## Abstract

The trend in reported case counts of invasive *Listeria monocytogenes* (*Lm*), a potentially severe food-borne disease, has been increasing since 2008. In 2015, 2,224 cases were reported in the European Union/European Economic Area (EU/EEA). We aimed to validate the microbiological and epidemiological aspects of an envisaged EU/EEA-wide surveillance system enhanced by routine whole genome sequencing (WGS). **Methods:** WGS and core genome multilocus sequence typing (cgMLST) were performed on isolates from 2,726 cases from 27 EU/EEA countries from 2010–15. **Results:** Quality controls for contamination, mixed *Lm* cultures and sequence quality classified nearly all isolates with a minimum average coverage of the genome of 55x as acceptable for analysis. Assessment of the cgMLST variation between six different pipelines revealed slightly less variation associated with assembly-based analysis compared to reads-based analysis. Epidemiological concordance, based on 152 isolates from 19 confirmed outbreaks and a cluster cutoff of seven allelic differences, was good (sensitivity > 95% for two cgMLST schemes of 1,748 and 1,701 loci each; PPV 58‒68%). The proportion of sporadic cases was slightly below 50%. Of remaining isolates, around one third were in clusters involving more than one country, often spanning several years. Detection of multi-country clusters was on average several months earlier when pooling the data at EU/EEA level, compared with first detection at national level. **Conclusions**: These findings provide a good basis for comprehensive EU/EEA-wide, WGS-enhanced surveillance of listeriosis. Time limits should not be used for hypothesis generation during outbreak investigations, but should be for analytical studies.

## Introduction

Invasive infection by *Listeria monocytogenes* (*Lm*) leads to relatively rare but serious food-borne disease mainly affecting elderly people, immunocompromised individuals and pregnant women. Clinical manifestations include sepsis and infection of the central nervous system, which can lead to lifelong sequelae or death [1,2]. Pregnancy-associated listeriosis can result in preterm birth, miscarriage or stillbirth [2,3]. In the European Union and European Economic Area (EU/EEA), 2,224 human cases of invasive listeriosis were reported in 2015, with an overall case fatality rate of 18.8% [4]. Reported numbers of cases of listeriosis suggest that the incidence of disease slightly increased over the period of 2010–15. The incubation period of listeriosis is usually 3 to 21 days, but can be as long as 67 days, depending on the clinical form of the disease [5]. Patients frequently have underlying conditions and/or are elderly, which limits the collection of exposure data in some cases. On epidemiological grounds, most cases are considered sporadic and detected outbreaks usually involve small numbers of patients, which limits statistical power in analytical epidemiological studies. As a result, most reported cases of listeriosis are difficult to link to a specific food product or food business operator.

*Lm* is able to form biofilms, grow at refrigeration temperature, high salt and nitrite concentrations, and can be resistant to disinfectants [6,7]. These properties contribute to its ability to persist and multiply in the food-processing environment and make it difficult to control. In the United States (US), a nationwide subtyping of *Lm* using pulsed-field gel electrophoresis (PFGE) was introduced in 1998. During the following 6 years, there was a more than fivefold increase in the number of outbreaks where a common food vehicle could be identified [8]. In the subsequent 10 years, the introduction of detailed food history gathering for all listeriosis patients resulted in a further increase in the number of solved outbreaks per year, as well as a reduction of the number of cases per outbreak [9].

PFGE is, however, time-consuming and difficult to standardise. In recent years, it has been demonstrated that whole genome sequence (WGS)-based subtyping can provide substantial additional discrimination and, consequently, can be of benefit to outbreak investigations [9-11]. Within the EU/EEA, listeriosis is one of the priority diseases for which supranational WGS-enhanced surveillance will be initiated in 2018 [12]. The work presented here supports the preparation for this surveillance system through a large-scale, retrospective, multi-centre study on *Lm* isolates from human cases from EU/EEA countries by covering the comparison and validation of analytical pipelines, the assessment of the epidemiological concordance of the results and the potential impact on public health [13]. The analytical pipelines are based on the gene-by-gene approach recommended by the PulseNet International global consortium [14-17].

## Methods

WGS was performed on a total of 2,726 *Lm* isolates from human cases from 27 EU/EEA countries and spanning years 2010–15 using Illumina MiSeq (Illumina, Inc., San Diego, California, US) 2x150 (n = 295), Illumina MiSeq 2x250 (n = 243), Illumina MiSeq 2x300 (n = 98), Illumina NextSeq 2x150 (n = 1,585) and Illumina HiSeq 2x100 (n = 422), as well as IonTorrent PGM (Thermo Fisher Scientific, Waltham, Maryland, US) (n = 83) platforms. Ten EU/EEA national public health laboratories (n = 1,069 isolates) and a commercial sequencing provider (n = 1,657 isolates) performed the sequencing.

### Trimming and de novo assembly

Trimming was performed on Illumina reads with Trimmomatic, before any further analysis. This included (i) removal of any adaptor sequences, (ii) removal of leading bases with PHRED < 25 (i.e. < 99.7% base call accuracy), (iii) removal of trailing bases with PHRED < 25, (iv) clipping of the remainder of the read when a sliding window of 20 bases has average PHRED < 25, and (v) removal of the entire read if length < 36 bases [18].

De novo assembly was performed with SPAdes and Velvet algorithms [19,20]. Spades 3.7.1 was run with BayesHammer read error correction and assembling mode with automatic determination of coverage cutoff, a minimum contig length of 300 nucleotides (nt) and with MismatchCorrector on. BWA-mem 0.7.12 was subsequently used to map all reads back to the SPAdes assembly, and consensus base calling was performed on the resulting alignment as an additional mismatch correction [21]. Velvet 1.1.04 was run with an automatic determination of the coverage cutoff, a minimum contig length of 300 bases using k-mers ranging from 59 to 69% of the average read length. The assembly with the highest N50 was retained. Bowtie2 was used to map all reads to this assembly, and consensus base calling was performed on the resulting alignment as mismatch correction [22].

### Allele calling

Allele calling was performed using two core genome multilocus sequence typing (cgMLST) schemes: the scheme of Moura et al. [10], as implemented in the BioNumerics (Applied Maths, Sint-Martens-Latem, Belgium) software (1,748 loci, available from http://bigsdb.pasteur.fr/listeria) and the scheme of Ruppitsch et al. [23], as implemented in the SeqSphere + (RIDOM, Münster, Germany) software (1,701 loci, available from http://www.cgmlst.org/ncs/schema/690488/). These schemes are further referred to as Moura CG and Ruppitsch CG. Both software applications were run with default parameters for allele calling, both based on either assemblies (assembly-based) or directly on reads (reads-based). In turn, each uses the basic local alignment search tool nucleotide (BLASTN) software to align assembled genomes to reference alleles: word size was 11, gap opening penalty 5 and gap extension penalty 2 [24]. Mismatch penalty was -3 and -1, and match reward 2 and 1 in Bionumerics 7.6.2 and SeqSphere +3.4.1, respectively. When more than one locus on the assembled genome matches a reference allele, such multiple allele calls are recorded, but no identifier is assigned. The direct reads-based allele calling in BioNumerics matches k-mers of size 35 from all known alleles against those of the reads, and considers the allele found if all its k-mers are present with the same number of occurrences. The SeqSphere + reads-based allele calling first maps the reads to reference genome NC_003210.1 using the BWA-SW software, and then produces a consensus sequence [21]. BLAST is subsequently used to align this sequence against the reference alleles for each locus, keeping only alignments that cover > 90% of the reference allele [24]. Alignments were verified for presence of start and stop codons and for a minimum of 70% (Moura CG) or 90% (Ruppitsch CG) nt sequence identity to reference alleles. For IonTorrent sequences, allele calling was only done by mapping reads first to the reference alleles and then again to the resulting consensus allele sequence, each time removing indels of 1 and 2 nt to specifically address this type of sequencing error.

To assess the impact of the different pipelines, i.e. combinations of input data (directly reads, SPAdes assembly or Velvet assembly) and scheme (Moura CG or Ruppitsch CG) on cgMLST analysis, two subsets of isolate pairs were used: pairs with AD ≤ 7 and pairs with AD ≤ 150. These correspond respectively to closely related isolates likely to share a common epidemiological link, and to sublineages where isolates are still likely to have common phenotypic properties that may be relevant, e.g. for source attribution [10,25].

### Quality control

Numeric quality indicators were defined to assess the following quality issues: contamination, *Lm* mixed cultures and sequence read quality. Two thresholds were determined per indicator to classify the result of the quality control (QC) as (i) acceptable ‘PASS’ or acceptable with a warning ‘WARN’ and (ii) not acceptable ‘FAIL’. The intermediate WARN level was added for practical application in public health, where a sequence may still be included if the time to re-sequence is too long compared with the time frame within which action should be taken. A final classification combining the results from all these quality controls is then made: ‘Accepted’ isolates have all QCs PASS, ‘AcceptedForOutbreak’ have some QCs WARN but none FAIL and ‘Rejected’ have at least one QC FAIL.

Contamination checks were performed using BLASTN by aligning the assembled genomes against fully closed genomes of other *Listeria* species (*L. ivanovii*, *L. marthii, L. seeligeri* and *L. welshimeri*) and species often encountered in laboratories for enteric diseases, including *Bacillus cereus*, *Campylobacter coli*, *C. fetus*, *C. hyointestinalis*, *C. jejuni*, *C. lari*, *C. upsaliensis*, *Clostridium botulinum*, *Cl. perfringens*, *Escherichia albertii*, *E. coli*, *E. fergusonii*, *Salmonella enterica*, *Shigella boydii*, *S. dysenteriae*, *S. flexneri*, *S. sonnei*, *Vibrio parahaemolyticus* and *Yersinia enterocolitica* [24]. Hits to the reference genome with at least 70% nt identity were kept and used to determine the proportion of the query genome covered by the reference genome. Mean and standard deviations (SD) of these proportions per reference genome were determined after one round of removing outliers with z-scores higher than 3 for the same genus and z > 6 for different genus references. Assemblies that were longer than 3.3 Mb were considered to be contaminated with another species as well. The contamination (CNTM) QC was set to WARN when a species of another genus was detected and to FAIL when other *Listeria* species were detected, since in the latter case it is likely that this will substantially interfere with allele calling.

Checks for *Lm* mixed cultures are much more difficult to assess than contamination with other species due to the inherent similarity between the strains. The QC used here was based on the multiple allele call results of cgMLST allele calling on SPAdes assembled genomes (see allele calling section). The number of core genome loci with multiple alleles (CGM) was used as an indicator that sequences are likely to originate from contaminated cultures. The CGM QC was set to WARN for isolates with CGM = 1, i.e. one locus with multiple allele calls, and to FAIL if CGM > 1.

Checks for sequence read quality were done based on the core genome coverage (CGC). This was defined as the proportion of core genome loci that were retrieved through allele calling. For each such locus, a biologically meaningful allele can therefore be found, including a start and a stop codon and a minimum similarity to curated reference alleles. Since DNA or sequencing quality issues are not expected to be biased towards core or accessory genome, the CGC values can be expected to be representative of the quality of the whole genome.

### Rarefaction analysis

Rarefaction curves were estimated for the effect of decreasing sampling fraction on both proportion of clustering vs sporadic isolates. This was also done to estimate the effect of decreasing CGC on allelic distances between closely related isolates, in order to assess to what extent CGC affects cgMLST analysis and cluster detection. The allelic distance (AD) between a pair of isolates was defined as the number of alleles across all loci in the scheme that are different, ignoring loci not present in either or both isolates. Estimations for all curves were performed with at least 100 random samples per point.

## Results

### Dataset

Table 1 presents the sequenced isolates by country and the respective proportion of officially reported listeriosis cases by year, retrieved from the European Centre for Disease Prevention and Control (ECDC) Surveillance Atlas of Infectious Diseases [4]. Overall in EU/EEA countries, 20–28% of all isolates from reported cases were sequenced for each year. This is a relatively uniform representativeness, though there was substantial variation between individual countries and also for some countries over the years.

**Table 1:** Number and proportion of sequenced isolates among all reported cases by country and year, *Listeria monocytogenes* whole genome sequencing study, European Union/European Economic Area, 2010‒2015 (n = 2,726)

Country	2010		2011		2012		2013		2014		2015		Total	
	n/N	%	n/N	%	n/N	%	n/N	%	n/N	%	n/N	%	n/N	%
Austria	32/34	94.1	21/26	80.8	1/36	2.8	17/36	47.2	42/49	85.7	33/38	86.8	**146/219**	**66.7**
Belgium	37/40	92.5	76/70	100.0	66/83	79.5	73/66	100.0	82/84	97.6	0/83	0.0	**334/426**	**78.4**
Bulgaria	4/4	100.0	4/4	100.0	7/10	70.0	0/3	0.0	6/10	60.0	2/5	40.0	**23/36**	**63.9**
Cyprus	1/1	100.0	2/2	100.0	0/1	0.0	0/1	0.0	0/0	NA	0/0	NA	**3/5**	**60.0**
Czech Republic	0/26	0.0	0/35	0.0	20/32	62.5	27/36	75.0	33/38	86.8	8/36	22.2	**88/203**	**43.3**
Germany	10/377	2.7	5/331	1.5	0/414	0.0	0/463	0.0	0/598	0.0	0/580	0.0	**15/2763**	**0.5**
Denmark	59/62	95.2	48/49	98.0	32/50	64.0	50/51	98.0	44/92	47.8	44/44	100.0	**277/348**	**79.6**
Estonia	0/5	0.0	0/3	0.0	0/3	0.0	0/2	0.0	4/1	100.0	10/11	90.9	**14/25**	**56.0**
Greece	0/10	0.0	0/10	0.0	0/11	0.0	3/10	30.0	3/10	30.0	13/31	41.9	**19/82**	**23.2**
Spain	17/129	13.2	20/91	22.0	6/109	5.5	32/140	22.9	13/161	8.1	0/206	0.0	**88/836**	**10.5**
Finland	21/71	29.6	10/43	23.3	10/61	16.4	13/61	21.3	22/65	33.8	44/46	95.7	**120/347**	**34.6**
France	15/312	4.8	20/282	7.1	0/348	0.0	0/369	0.0	0/374	0.0	0/410	0.0	**35/2,095**	**1.7**
Hungary	11/20	55.0	1/11	9.1	2/13	15.4	1/24	4.2	5/39	12.8	0/37	0.0	**20/144**	**13.9**
Ireland	4/10	40.0	5/7	71.4	10/11	90.9	6/8	75.0	13/15	86.7	16/19	84.2	**54/70**	**77.1**
Iceland	1/1	100.0	1/2	50.0	4/4	100.0	1/1	100.0	4/4	100.0	0/0	NA	**11/12**	**91.7**
Italy	14/157	8.9	23/129	17.8	8/112	7.1	18/143	12.6	15/132	11.4	28/153	18.3	**106/826**	**12.8**
Lithuania	4/5	80.0	2/6	33.3	6/8	75.0	1/6	16.7	8/7	100.0	0/5	0.0	**21/37**	**56.8**
Luxembourg	0/0	NA	1/2	50.0	2/2	100.0	1/2	50.0	5/5	100.0	0/0	NA	**9/11**	**81.8**
The Netherlands	61/72	84.7	74/87	85.1	62/73	84.9	53/72	73.6	67/90	74.4	46/71	64.8	**363/465**	**78.1**
Norway	21/22	95.5	20/21	95.2	27/30	90.0	18/21	85.7	26/29	89.7	18/18	100.0	**130/141**	**92.2**
Poland	15/59	25.4	28/62	45.2	55/54	100.0	41/58	70.7	60/87	69.0	34/70	48.6	**233/390**	**59.7**
Portugal	0/0	NA	1/1	100.0	4/4	100.0	12/12	100.0	18/18	100.0	24/28	85.7	**59/63**	**93.7**
Romania	4/6	66.7	5/1	100.0	2/11	18.2	1/9	11.1	2/5	40.0	7/12	58.3	**21/44**	**47.7**
Sweden	53/63	84.1	34/56	60.7	0/72	0.0	70/93	75.3	28/125	22.4	51/88	58.0	**236/497**	**47.5**
Slovenia	12/11	100.0	4/5	80.0	4/7	57.1	17/16	100.0	19/18	100.0	12/13	92.3	**68/70**	**97.1**
Slovakia	0/5	0.0	0/31	0.0	0/11	0.0	0/16	0.0	0/29	0.0	3/18	16.7	**3/110**	**2.7**
United Kingdom	18/176	10.2	23/164	14.0	16/183	8.7	65/192	33.9	40/201	19.9	68/186	36.6	**230/1,102**	**20.9**
Total EU/EEA	414/16,86	24.6	428/1,540	27.8	344/1,760	19.5	520/1,917	27.1	559/2,294	24.4	461/2,222	20.7	**2,726/11,419**	**23.9**

EU/EEA: European Union/European Economic Area; NA: not applicable (denotes the absence of any reported listeriosis cases).

### Sequence data quality

The contamination (CNTM) QC detected three *L. ivanovii* isolates (0.1%; CNTM result set to FAIL) and 31 isolates that contained bacterial DNA from genera other than *Listeria* spp. (1.1%; CNTM set to WARN). The mixed *Lm* culture (CGM) QC detected two isolates with CGM = 1 (WARN) and six with CGM > 1 (ranging 2–21; FAIL). Of these eight isolates, five also had CNTM WARN, due to assembly lengths > 3.3 Mb.

**Figure 1:**
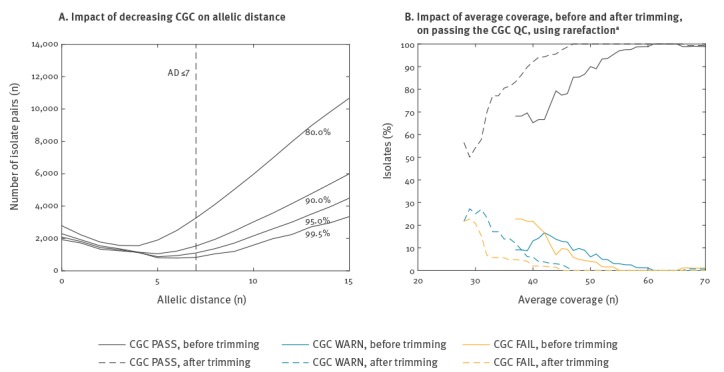
Sequence data quality control based on core genome coverage (CGC), (A) Impact of decreasing CGC on allelic distance (n = 2,664), (B) Impact of average coverage, before and after trimming, on passing the CGC quality control, using rarefaction^a^ (n = 2,609), *Listeria monocytogenes* whole genome sequencing study, European Union/European Economic Area, 2010‒2015

AD: allelic distance; CGC: core genome coverage; QC: quality control.

^a^A sliding window of ± 5x coverage was used, with a minimum number of 10 observations per window.

The final quality issue that was investigated was poor quality of the sequence reads using the CGC QC. Rarefaction analysis of artificially reduced CGC by randomly deleting loci was performed using Moura CG, SPAdes assembly and including only isolates with actual CGC ≥ 95%, CNTM PASS and CGM PASS (n = 2,664). This showed that a reduction from 99.5% on average to 95.0%, 90.0% and 80.0% led to an increase in the number of isolate pairs with AD ≤ 7 by 9.9%, 26.1% and 89.5%, respectively (Figure 1a). Based on this, and the fact that most of these additional pairs remained in the AD 5–7 region, we selected PASS ≥ 95% and FAIL < 90% (WARN between 90–95%) as thresholds for this QC. A total of 2,692 (98.8%) isolates passed, 16 (0.6%) passed with a warning and 18 (0.7%) failed.

Figure 1b shows the impact of the average coverage on the CGC QC, with a clear improvement in the proportion of acceptable isolates as coverage increases up to around 55x before trimming and 45x after trimming. Only CNTM PASS and CGM PASS isolates were included and 83 IonTorrent sequences (3.0%) were excluded from this analysis in order to not mix results from two different platforms, leaving n = 2,609 isolates for the analysis. In contrast, the corresponding curve for a stricter threshold of 97.5% for acceptable CGC reaches this plateau only at around 80x coverage before trimming (data not shown).

In summary, for our dataset, 22 isolates (0.8%) failed at least one QC and were classified as ‘Rejected’. No association was found between the sequencing platform used and failing the CGC QC that assesses sequence quality. A total of 40 isolates (1.5%) passed at least one QC with a warning but failed none, and these were classified as ‘AcceptedForOutbreak’. The remaining 2,664 (97.7%) passed all three QCs and were classified as ‘Accepted’, and only these were used for subsequent analyses on analytical and epidemiological validation.

### Analytical validation

The variation introduced by using different input data (directly reads, SPAdes assemblies or Velvet assemblies) and different schemes (Moura CG or Ruppitsch CG) is summarised in Table 2, based primarily on the difference in AD (Δ_AD_), between the most relevant pairs of methods. Other pairs gave near-identical results (data not shown). The Δ_AD_ between SPAdes and Velvet was small, with a mean of -0.19 vs -0.29 depending on the scheme for AD ≤ 7. Velvet had in 4,477 of 50,083 (8.9%) pairs at least one additional allelic difference compared with SPAdes among loci present in both assemblies for the AD ≤ 150 set, with 83 (0.2%) for the reverse case. The Velvet assembly length was also shorter in 667 of 1,023 (65.2%) cases where a Velvet assembly was performed. Further assembly-based analyses were therefore based on SPAdes, though results were near-equivalent. The Δ_AD_ between SPAdes-based and reads-based allele calling was slightly skewed towards more differences based on reads, even though less loci were detected from the reads. Reads-based allele calling therefore seems prone to more variation than assembly-based allele calling. The difference between the two core genome schemes was larger than that between reads and assembly-based allele calling.

**Table 2:** Independent contribution of source data and scheme to variation in allelic distance between different allele calling methods, *Listeria monocytogenes* whole genome sequencing study, European Union/European Economic Area, 2010‒2015

Method 1	Method 2	Pairs for comparison AD ≤ 7 (n) vs AD ≤ 150 (n)	Mean CGC comparison method 1 (%) vs 2 (%)	Δ_AD_^a^, subset AD ≤ 7		Δ_AD_^a^, subset AD ≤ 150	
				Mean	Range^b^	Mean	Range^a^
SPAdes + Moura CG	Velvet + Moura CG	313 vs 24,196	99.5 vs 99.7	- 0.2	- 2 to 0	- 0.8	- 5 to 1
SPAdes + Ruppitsch CG	Velvet + Ruppitsch CG	1,229 vs 50,233	99.2 vs 99.4	- 0.3	- 2 to 0	- 0.6	- 4 to 1
SPAdes + Moura CG	Reads + Moura CG	2,780 vs 135,365	99.5 vs 99.1	- 1.1	- 5 to 1	- 0.6	- 4 to 3
SPAdes + Ruppitsch CG	Reads + Ruppitsch CG	1,237 vs 50,881	99.2 vs 98.9	- 1.2	- 4 to 0	- 1.1	- 5 to 2
Velvet + Moura CG	Reads + Moura CG	148 vs 9,040	99.7 vs 99.1	- 0.6	- 2 to 1	- 0.7	- 5 to 5
Velvet + Ruppitsch CG	Reads + Ruppitsch CG	1,193 vs 50,381	99.4 vs 98.9	- 0.9	- 4 to 1	- 0.6	- 4 to 2
SPAdes + Moura CG	SPAdes + Ruppitsch CG	5,255 vs 230,478	99.5 vs 99.2	0.6	- 5 to 3	- 1.9	- 14 to 9
Velvet + Moura CG	Velvet + Ruppitsch CG	337 vs 24,196	99.7 vs 99.4	- 0.0	- 4 to 4	- 1.5	- 12 to 8
Reads + Moura CG	Reads + Ruppitsch CG	549 vs 19,721	99.1 vs 98.9	0.2	- 3 to 4	- 0.8	- 13 to 10

AD: allelic distance; CG: core genome; CGC: core genome coverage.

^a^Δ_AD_ is calculated as AD of method 1 minus that of method 2

^b^Range represents the smallest possible Δ_AD_ range for ≥ 95% of the isolate pairs.

### Epidemiological validation

**Figure 2:**
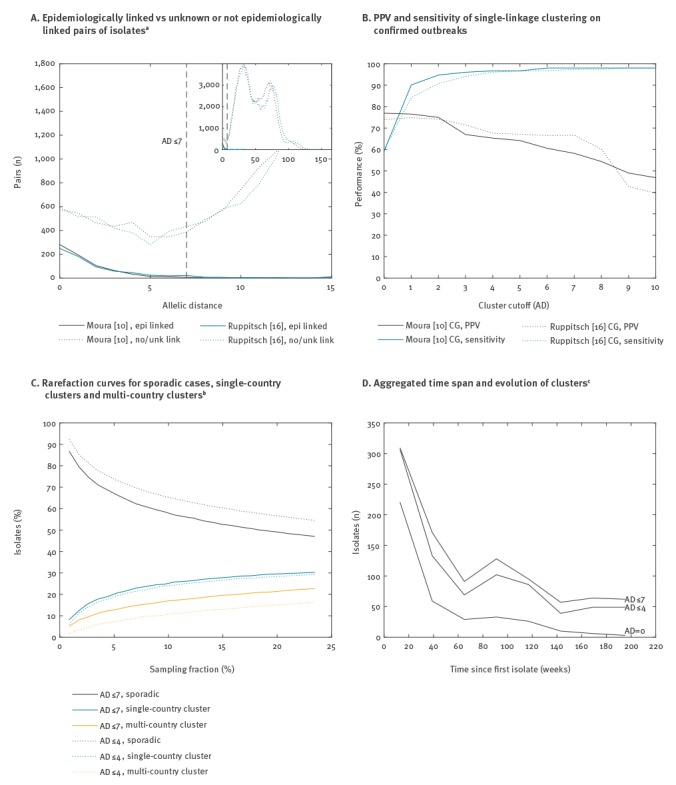
Concordance between cgMLST and epidemiological results for (A) Epidemiologically linked vs unknown or not epidemiologically linked pairs of isolates^a^, (B) Positive predictive value and sensitivity of single-linkage clustering on confirmed outbreaks, (C) Rarefaction curves for sporadic cases, single-country clusters and multi-country clusters^b^, (D) Aggregated time span and evolution of clusters^c^; *Listeria monocytogenes* whole genome sequencing study European Union/European Economic Area, 2010‒2015 (n = 2,664)

AD: allelic distance; CG: core genome; epi link: epidemiologically linked; no/unk link: unknown or not epidemiologically linked; PPV: positive predictive value.

^a^The inset shows all pairs up to AD ≤ 150.

^b^Cutoff AD ≤ 7.

^c^Aggregated by setting the first isolate of each cluster at t = 0 and using bins of 26 weeks, i.e. half a year.

Our dataset contained 19 confirmed outbreaks from 10 EU/EEA countries with a median of 6.5 cases (range: 3‒19), plus two mother-child pairs, encompassing 152 isolates. These outbreaks were both microbiologically and epidemiologically confirmed by the respective national authorities, but confirmation was done using microbiological typing methods other than WGS. The outbreaks therefore represent an independent test set for evaluating epidemiological concordance of WGS. The number of corresponding epidemiologically linked pairs decreased with increasing AD (Figure 2a), confirming that low cgMLST AD strongly correlates with epidemiological linkage. This pattern was similar between the two cgMLST schemes, as well as between allele calling approaches (data not shown).

Cluster detection was done using single linkage clustering and subsequently applying a cluster cutoff to the tree. For each of the confirmed outbreaks, only the cluster that had the most isolates in common with the outbreak was considered to be the predicted outbreak. Based on this, true positive, false positive and false negative isolates were enumerated and summed across the outbreaks. Figure 2b shows both the resulting positive predictive value (PPV) and the sensitivity as a function of the cluster cutoff. As for isolate pairs, both cgMLST schemes have similar performance and, in both cases, sensitivity reaches a plateau of > 95% at AD ≤ 4. PPV was 65.3% vs 67.6% for Moura CG vs Ruppitsch CG at AD ≤ 4 and 58.2% vs 66.7% at AD ≤ 7. There were, for example, 28 vs 33 (Moura CG vs Ruppitsch CG) isolates matching with AD =  0 to one of the 152 isolates in confirmed outbreaks, indicating that the real PPV and sensitivity may still be higher since all of these isolates would also have been classified as true positives.

### Microbiological cluster detection

The proportion of sporadic cases, i.e. cases that do not cluster with any other, was 47.0% vs 47.8% using AD ≤ 7 and 54.5% vs 55.2% using AD ≤ 4. Figure 2c shows the effect of sampling fraction on the proportion of sporadic cases, using rarefaction and the Moura CG (similar results for Ruppitsch CG, data not shown). The isolates that clustered were further stratified by belonging to either a single- or a multi-country cluster.

Table 3 further stratifies the number of clusters based on size, cluster cutoff and single- vs multi-country. Among the multi-country clusters, a distinction is made between clusters in which each country has only one isolate, and that can thus only be detected at EU/EEA level, and clusters in which at least one country has more than one isolate. The latter can be detected first either at national level or at EU/EEA level, depending on whether the second isolate of any involved country is also the second isolate overall. The average number of days that EU/EEA level detection for clusters of more than five isolates was earlier than national detection was at least 75 (AD ≤ 4) and at most 154 (AD ≤ 7). This indicates that, in terms of timeliness of detection, there can be a substantial gain by pooling data at the EU/EEA level.

**Table 3:** Single- and multi-country clusters, *Listeria monocytogenes* whole genome sequencing study, European Union/European Economic Area, 2010‒2015

Cluster size (n)	Total (n)	Single country		Multi-country				
		N	%	Each country one isolate		Some countries with more than one isolate		Average time gained through detection at EU-level (days)
				N	%	N	%	
**Cutoff (AD) ≤ 4 (n = 317)**								
2	172	132	76.7	40	23.3	NA	NA	NA
3–5	98	60	61.2	4	4.1	34	34.7	144
> 5	47	25	53.2	0	0.0	22	46.8	75
**Cutoff (AD) ≤ 7 (n = 331)**								
2	166	125	75.3	41	24.7	NA	NA	NA
3–5	109	68	62.4	4	3.7	37	33.9	136
> 5	56	25	44.6	0	0.0	31	55.4	154

AD: allelic distance; EU: European Union; NA: not applicable.

Finally, the 6-year time span of this dataset also allows examination of the evolution of clusters over time. Figure 2d shows the aggregated number of isolates over a period of nearly 4 years belonging to the same cluster, with the first isolate in the cluster set at t = 0. These clusters were created using single linkage clustering and different AD cutoffs of 0, ≤ 4 and ≤ 7 to assess the impact of the cutoff on cluster duration and size. For our dataset, and using cutoff AD ≤ 7, clusters of sizes two, three and larger than three had a median duration of 16, 78 and 112 weeks, respectively, for single-country clusters. For multi-country clusters, this increased to 34, 84 and 181 weeks, respectively.

## Discussion

In this large multi-country study, we examined both the analytical and general epidemiological aspects of surveillance for human *Lm* infections using WGS. We identified CGC as the one main quality indicator that detected most of the quality issues in WGS data. CGM and contamination checks were also investigated, but these identified few additional issues, although further research on the extent and impact of contaminations and mixed cultures is warranted. The low occurrence of contamination indicates a high level of quality among participating laboratories. Quality control criteria were established to classify isolates as ‘Accepted’, ‘AcceptedForOutbreak’ and ‘Rejected’.

We conclude that the isolate sequences classified as ‘Accepted’ by the criteria outlined here are of sufficient quality to be a reliable basis for supporting epidemiological investigations and, in particular, cluster detection and outbreak investigations, also in an international setting. Although 2.3% of the isolates did not reach this level of quality, there is a clear correlation with the average coverage up to around 55x before trimming and 45x after trimming for the Illumina platform. This would therefore be a recommended minimum coverage for this platform for laboratories getting started with WGS for *Lm* to ensure the reliability of the data and downstream analyses, consistent with previous reports [10]. It is also possible that further improvements through, for example, internal and external quality assessment exercises and new sequencing technology, could result in lower coverage yielding the same quality.

Based on the comparison of different allele calling methods, it can be concluded that assembly-based allele calling outperforms reads-based allele calling with the methods used here. More loci were detected, which increased typeability, and the average distances between isolates were slightly smaller. Velvet including k-mer optimisation performed slightly worse than SPAdes, but both produced near-equivalent results. This provides an important opportunity to simplify the cooperation between national public health institutes and laboratories on WGS-enhanced surveillance, thereby enhancing their ability to respond to listeriosis cross-border clusters and outbreaks. Provided that validated assemblers are used to assure data reproducibility [10], and in the absence of a globally accepted unique strain nomenclature to classify isolates, it is sufficient for cgMLST analysis to share assembled genomes rather than sequence read data, which often require an additional separate system and workload. When further confirmation is required, e.g. when deciding on control measures in multi-country outbreaks, it may still be necessary to share sequence reads, e.g. for SNP analysis or to verify the analyses, but this is likely only for a small minority of the cases. For communication on outbreaks or detected clusters, it is important to keep in mind that the individual differences in ADs between Moura and Ruppitsch CG schemes can be relatively large, due to the different set of loci used, since only 1,261 loci are common to both schemes [10]. This may impact the formulation of outbreak case definitions, which should always specify what cgMLST scheme(s) are used, as well as their total number of loci, minimum WGS quality criteria and cutoffs.

The AD ≤ 7 cutoff defined in Moura et al. is confirmed as useful for cluster detection for both the Moura and the Ruppitsch CG [10,23]. A more stringent cluster cutoff of AD ≤ 4 in combination with single linkage clustering may be considered for identifying isolates with more compelling microbiological evidence of being part of the same outbreak. A second higher cutoff for weaker microbiological evidence could be applied as well, in order to define, for example, confirmed vs probable cases in an outbreak case definition. More confirmed outbreaks are needed to have a more accurate estimation of PPV and sensitivity as a function of cluster cutoff. Since confirmed outbreaks were delineated at national level only and using less discriminatory methods than cgMLST, there may be additional isolates in the dataset that are actually part of one of the confirmed outbreaks, leading to underestimation of PPV and possibly sensitivity. More confirmed outbreaks are also needed to understand why epidemiologically linked isolates could have substantially more allele differences than the cutoff, as described earlier [9,10]. One possibility for microbiological diversity in an epidemiological cluster is that a single source or environmental niche may be occupied by more than one strain. Another is that specific sublineages may have higher average mutation rates, and if sufficient evidence is available to justify it, sublineage-specific cutoffs could be defined. Reliability of establishing microbiological relatedness is crucial in practice for public health, since it increases the power of analytical studies on exposure data. In addition, though unlikely for *Lm* given its low incidence and high severity, when exposure data cannot be gathered for all cases, it would help in selecting the cases for which to gather exposure data.

The aggregated curves of number of cases as a function of time were also similar regardless of the cutoff used (Figure 1d), indicating that clusters of closely related isolates can persist for several years, as previously reported [11,26-28]. Such a long time span of microbiological clusters can be expected given the microbiological properties of *Lm*, such as a slow mutation rate of around 1 SNP per year and the ability to form biofilms that are difficult to eradicate in food processing environments [6,7,10,29]. This is important to take into account, especially for control measures, since knowledge of retrospective isolates, whether from human cases or food sources, may still inform investigations of new cases [9,11,27,30]. Therefore, an additional restriction on time for defining microbiological clusters of *Lm* does not seem warranted for descriptive epidemiology and hypothesis generation, while it is likely still required for analytical epidemiological studies.

Around a third of the clusters found involved more than one country, and for clusters of more than five isolates this increased to around half. This can be expected given the international nature of food trade, and indicates that there is potential for a substantial added public health value of introducing EU/EEA-wide, WGS-enhanced surveillance of listeriosis. Among other benefits, it may lead to earlier detection of clusters, which in our dataset was on average several months for multi-country clusters. At the same time, the molecular typing results must also be combined with epidemiological and food exposure investigations. Given the sometimes-long incubation period of listeriosis, the low number of cases and the severity of the disease, food exposure data should ideally be collected for all cases, without additional waiting for typing results, as presently done in, for example, Denmark, France and the US. It should also be complemented by WGS typing of officially sampled food isolates, and be combined with a joint analysis of the microbiological data to detect potential links between human cases and food items [11,31]. The successful implementation of control measures for multi-country persistent clusters also requires cross-border investigations and intense collaboration of public health and food safety authorities and laboratories.

Assuming that all isolates clustering within the cutoff are epidemiologically linked, there is a substantial amount of apparently sporadic cases [32]. Based on the rarefaction analysis, around half of the cases in this study were sporadic cases. However, this proportion is likely to be slightly lower for comprehensive sampling. Any bias in the selection of isolates in the different countries, with respect to their likelihood of being part of an outbreak, may also affect this number. It is likely that some proportion of cases is truly sporadic in the sense that they are isolated cases related to individual- or household-level food preparation or storage practices, rather than to the microbiological quality of the food at the time of purchase [33]. In general, these cases can only be addressed through preventive measures such as public education, rather than control measures for food business operators. Further research is needed to determine to what extent specific populations face an increased risk, as well as how preventive measures such as microbiological criteria for food could be improved. Finally, similar studies could be useful for other pathogens, in particular *Salmonella enterica,* Shiga toxin-/verocytotoxin-producing *Escherichia coli* (STEC/VTEC) and *Campylobacter* spp., to establish appropriate sequence quality criteria, to assess epidemiological concordance as a function of the analysis method and to estimate the added value of national and EU/EEA-wide WGS-enhanced surveillance.

## Notes

**European *Listeria* WGS typing group:** Wesley Mattheus (Wetenschappelijk Instituut Volksgezondheid, Brussels, Belgium), Iva Christova (National Center of Infectious and Parasitic Diseases, Sofia, Bulgaria), Panayiota Maikanti-Charalampous (Nicosia General Hospital, Nicosia, Cyprus), Renáta Karpíšková (Výzkumný ústav veterinárního lékařství, Brno, Czech Republic), Sven Halbedel (Robert Koch-Institut, Wernigerode, Germany), Eva Møller Nielsen (Statens Serum Institut, Copenhagen, Denmark), Marliin Koolmeister (Terviseamet, Tallinn, Estonia), Georgia Mandilara (National Reference Center for Salmonella, Shigella, VTEC and Listeria, Vari Attiki, Greece), Raquel Abad Torreblanca (Instituto de Salud Carlos III, Madrid, Spain), Saara Salmenlinna (National Institute for Health and Welfare, Helsinki, Finland), Marc Lecuit (Institut Pasteur, National Reference Center and WHO Collaborating Center Listeria, Biology of Infection Unit, Inserm U1117, and Paris Descartes University, Paris, France), Alexandre Leclercq (Institut Pasteur, National Reference Center and WHO Collaborating Center Listeria, Paris, France), Ivelina Damjanova (Országos Epidemiológia Központ, Budapest, Hungary), Niall Delappe (University Hospital Galway, Galway, Ireland), Guðrún Sigmundsdóttir (Landspítali University Hospital, Reykjavik, Iceland), Antonietta Gattuso (Instituto Superiore di Sanità, Rome, Italy), Algirdas Griškevičius (National Public Health Surveillance Laboratory, Vilnius, Lithuania), Catherine Ragimbeau (Laboratoire national de santé, Dudelange, Luxembourg), Eelco Franz (Rijksinstituut voor Volksgezondheid en Milieu, Bilthoven, Netherlands), Lin Thorstensen Brandal (Norwegian Institute of Public Health, Oslo, Norway), Alicja Kuch (National Medicines Institute, Warsaw, Poland), Vítor Borges (Instituto Nacional de Saúde Doutor Ricardo Jorge, Lisbon, Portugal), Dana Caplan (Cantacuzino National Institute for Research, Bucharest, Romania), Cecilia Jernberg (The Public Health Agency of Sweden, Stockholm, Sweden), Erik Alm (The Public Health Agency of Sweden, Stockholm, Sweden), Marija Trkov (National Laboratory of Health, Environment and Food, Ljubljana, Slovenia), Eva Tkacova (National Reference Laboratory for Listeriosis, Kosice, Slovakia), Kathie Grant (Public Health England, London, United Kingdom).
